# Beyond a single scan: physicochemical modification of enamel subjected to multiple Nd:YAG laser irradiations in a preventive context

**DOI:** 10.1007/s10103-026-05084-z

**Published:** 2026-11-09

**Authors:** Júlia Guerra Cavalero, Daniela Fátima Teixeira Silva, Carlos de Paula Eduardo, Denise Maria Zezell

**Affiliations:** 1 Center for Lasers and Applications, Instituto de Pesquisas Energéticas e Nucleares, São Paulo, Brazil; 2 Universidade de São Paulo, São Paulo, Brazil; 3 Faculdade de Odontologia da USP (FOUSP), Universidade de São Paulo, São Paulo, Brazil

**Keywords:** Nd:YAG laser, Dental enamel, Acidulated phosphate fluoride, Preventive dentistry, SEM, Chemical composition

## Abstract

To investigate the surface modification (changes in roughness, morphology, and chemical composition) of human enamel subjected to multiple Nd:YAG laser irradiations over a photoabsorber, with and without the subsequent application of acidulated phosphate fluoride (APF), in a caries control protocol. 150 enamel blocks (4 × 4 mm) from human third molars were randomly distributed into 10 groups based on surface microhardness: control groups (CN, CF) and irradiated groups subjected to 1, 2, 3, or 4 successive scans. A pulsed Nd:YAG laser (1064 nm, 65 mJ/pulse, 10 Hz, 51.7 J/cm²) irradiated the surfaces using a motorized stage with calculated speed for partial pulse overlap. An aqueous coal powder suspension (equal parts of ultrapure water and 99% ethanol) was applied before each scan. APF 1.23% was applied post-irradiation for the fluoridated groups. The surfaces were analyzed by optical profilometry (Sa), Scanning Electron Microscopy (SEM), and Attenuated Total Reflection Fourier Transform Infrared Spectroscopy (ATR-FTIR). Qualitative analysis of profilometry and SEM images revealed that one or two laser scans resulted in unaltered areas and spaces of non-melted enamel between pulses. Groups irradiated three or four times achieved more uniform areas of surface melting, also exhibiting significantly higher surface roughness. ATR-FTIR analysis, guided by multivariate models and semi-quantitative analysis, showed indications that laser irradiation promoted thermal decarbonation of the enamel and caused the narrowing of phosphate bands. One or two Nd:YAG laser scans were insufficient to modify the entire enamel surface. Successive irradiations with reapplication of the photoabsorber must be performed to ensure that the whole targeted area is modified, despite the localized increase in surface roughness.

## Introduction

Dental caries remains a global public health challenge [1, 2]. Although the introduction of fluoride has drastically reduced its incidence, the multifactorial nature of the disease prevents this from being a definitive and universal solution [3]. Therefore, the use of high-power lasers has been investigated for over five decades as an adjunctive preventive method, aiming to promote structural and compositional changes in the enamel that make it more resistant to demineralization [4, 5].

The Nd:YAG laser (1064 nm) showed promising laboratory and clinical results [6, 7]. The evolution of scientific thought regarding its mechanism of action transitioned from the initial hypothesis of decreased permeability by “sealing” to the current understanding of a complex physical-chemical reorganization [8]. Studies have demonstrated that the thermal increase caused by the laser leads to the loss of water and carbonate, an increase in crystal diameter (micro), an increase in surface area (macro) due to melting, and greater interaction with fluoride ions, forming a larger CaF_2_ reservoir [9–11].

Because the Nd:YAG wavelength is poorly absorbed by sound enamel, the literature has established the need to use a photoabsorber (such as India ink or an aqueous coal suspension) on the surface prior to irradiation [10, 12, 13]. This strategy concentrates the absorption of infrared radiation on the tissue surface, promoting superficial melting (above 1200 °C) and ensuring pulpal safety (negligible intrapulpal heating) [8, 14, 15].

Based on these premises, a randomized clinical trial with 33 patients demonstrated an expressive 39.2% reduction in caries incidence after 12 months (compared to unlased control group) using Nd:YAG laser associated with acidulated phosphate fluoride (APF) gel [7]. This clinical preventive protocol was based on three consecutive irradiations (scans), with reapplication of the photoabsorber, according to previous evidence [10]. However, despite the clinical relevance of these findings, the use of the laser has not been consolidated in the preventive clinical routine. A well-regarded literature review [8] summarized the main reason for this limitation: a lack of methodological standardization across studies. With rare exceptions [16], most recent literature has diverged in essential parameters, greatly varying energy density [17, 18] and neglecting the irradiation process by applying the laser without a photoabsorber [17–21] or performing a single manual scan [17–26].

Such simplification hides a fundamental physical incongruence. The Nd:YAG laser light, being collimated and delivered through optical fibers with diameters on the order of 300 to 400 μm, results in pulses with highly localized thermal effects. The photoabsorbing substance is mostly vaporized in the very first pulses [10]. Therefore, assuming that the preventive success of the therapy depends on the structural transformation of the entire target surface, it is physically unlikely that a single irradiation can alter the whole enamel homogeneously. Manual irradiation results in spaced pulses without overlap, so that the outcome is a surface changed in a localized and heterogeneous manner [16, 27]. That is, not only the laser parameters but also the irradiation technique must directly affect the treatment outcomes, contributing to the dilution of evidence supporting Nd:YAG laser in caries prevention in the current literature.

Although physical evidence indicates that repeating the laser scans promotes a more uniform distribution of the alteration, there are few published, structured results elucidating the modification generated by repeated irradiations. In light of this gap, the present study investigated the topographic, morphological, and chemical differences of human enamel irradiated sequentially from one to four times with an Nd:YAG laser. To fully characterize these alterations, a multiparametric approach is required. Optical profilometry provides non-destructive 3D topographical quantification, allowing the spatial mapping of surface roughness. Complementarily, Scanning Electron Microscopy (SEM) offers high-resolution imaging to qualitatively assess the morphological patterns of melting, resolidification, and adjacent thermal impact. Finally, Attenuated Total Reflection Fourier Transform Infrared Spectroscopy (ATR-FTIR) explores the vibrational modes of the tissue’s components, tracking thermal decarbonation and crystallographic reorganizations within the enamel lattice. Together, these techniques link macrostructural topography with microstructural and chemical modifications. The null hypothesis was that a single irradiation is incapable of changing the entire target surface, making the repetition of scans (with photoabsorber reapplication) necessary to ensure comprehensive surface modification.

## Materials and methods

### Study design and sample preparation

This in vitro, randomized, and controlled study used sound human third molars to prepare 150 samples. Enamel blocks (4 × 4 × 2 mm) were obtained by sectioning the proximal, buccal, and lingual surfaces using a precision saw (Isomet, Buehler, Lake Bluff, IL, USA). Subsequently, they were embedded in self-curing acrylic resin inside PVC cylinders, leaving only the enamel surface (4 × 4 mm) exposed for flattening and polishing in a semi-automatic polisher (EcoMet 250/300, Buehler, Lake Bluff, IL, USA) with progressive abrasive papers (up to 4000 grit).

Initial surface microhardness (Knoop, 245.2 mN, 5 s; HMZ-2000, Shimadzu, Kyoto, Japan) was measured at 3 points on the surface to standardize the samples. Specimens with mean values more than 10% above or below the overall mean (443.78 KHN) were excluded, and the remaining samples were stratified according to their KHN values and randomly allocated into 10 experimental groups (with mean *n* = 15) to ensure baseline homogeneity among the groups (overall mean = 448.49 ± 0.33 KHN). The ten experimental groups were defined by the number of sequential laser scans from one to four times, without (L1, L2, L3, L4) or with the subsequent application of APF (L1F, L2F, L3F, L4F), in addition to the negative control (CN) and fluoride-only (CF) groups.

### Laser irradiation and fluoride application

Irradiations were performed with a pulsed Nd:YAG laser (1064 nm; Smarty-A10, DEKA M.E.L.A., Calenzano, Italy) using a 400 μm diameter optical fiber. Because this wavelength is poorly absorbed by sound enamel, an aqueous photoabsorber suspension (coal powder mixed with equal parts of ultrapure water and 99% ethanol) was applied using a small brush and dried before each laser scan in all irradiated groups.

To ensure strict reproducibility and eliminate the irradiation variability inherent to manual operation, the handpiece with the optical fiber was fixed perpendicularly at a distance of 1 mm from the sample flat surface, while the sample was attached to and moved by the motorized stage. The power was measured with a power meter (FieldMaster GS, Coherent, Santa Clara, CA, USA) before starting the procedures and after every 10 irradiations. The irradiation parameters were: *energy per pulse*: 65 mJ (average power ~0.65 W); *frequency*: 10 Hz; *pulse duration*: 100 µs; *scan speed (motorized stage)*: 2.52 mm/s, calculated for a 37% pulse overlap; *estimated energy density at the fiber end*: 51.7 J/cm²; *cooling*: without external cooling.

The laser protocols were followed by standardized low-speed brushing and ultrasonic cleaning to remove coal residues, and after that 0.1 ml of APF gel (1.23% F⁻; LysFluor, Lysanda, São Paulo, SP, Brazil) was topically applied to the “F” groups for four minutes. After this time, the excess was removed with absorbent paper, and the samples remained undisturbed for 30 min before being washed with ultrapure water, simulating the manufacturer’s recommendations.

### Analytical methods

#### Optical profilometry

The quantitative evaluation of roughness (Sa, area roughness) was performed non-destructively with a 3D optical profilometer (ZeGage, Zygo Corporation, Middlefield, CT, USA; Zemetrics ZeMaps software). A 2.25 mm² area near the center of each specimen (*n* = 15/group) was scanned using a 5.5x objective (100 μm scan length). The 2D and 3D images generated by the profilometer were evaluated qualitatively to understand the spatial distribution of the pulses and their alteration on the surface. The areal surface roughness (Sa) was calculated using the software’s default surface texture parameters (ISO 25178). A standard tilt form removal was applied to correct sample leveling, and no additional waviness cutoff filters were applied (Cutoff Period: Off).

#### Scanning electron microscopy (SEM)

This characterization was performed on 3 specimens per group. The samples were dehydrated in an ascending ethanol concentration (50% to 100%) to minimize vacuum-induced artifacts in the structure. The surfaces were analyzed in a benchtop SEM (TM3000, Hitachi, Tokyo, Japan) at 5 kV, with images captured at progressive magnifications (from 40x to 2,000x) to evaluate both macro and microscopic fusion patterns.

#### Attenuated total reflection Fourier transform infrared spectroscopy (ATR-FTIR)

Chemical/crystallographic modifications were evaluated (*n* = 12/group) in a Cary Series FTIR spectrometer (Agilent Technologies, Santa Clara, CA, USA) equipped with a diamond ATR crystal. A layer of Nujol was applied to reduce the air gap at the sample/crystal interface (index matching). Spectra were acquired from 4000 to 400 cm⁻¹ with a resolution of 4 cm⁻¹, using 128 background scans and 64 sample scans.

### Statistical analysis

Data tabulation, processing, and statistical inference were automated using customized Python scripts executed in the Google Colaboratory (Colab) environment, ensuring full analytical reproducibility. For profilometry (Sa) and areas under the curve of ATR-FTIR (from the most influential bands in group separation according to multivariate analysis - LDA), since the normal distribution assumptions were not met by all groups, the data underwent a Yeo-Johnson transformation prior to two-way ANOVA (laser and fluoride) followed by Tukey’s post-hoc test. The adopted significance level was 5%. Effect sizes were reported using partial eta-squared (η^2^_p_) and interpreted according to established guidelines, where η^2^_p_ *≥* 0.14 indicates a large effect size, representing the proportion of variance explained by the studied factors.

## Results

### Optical profilometry (topography and roughness)

**Fig. 1:**
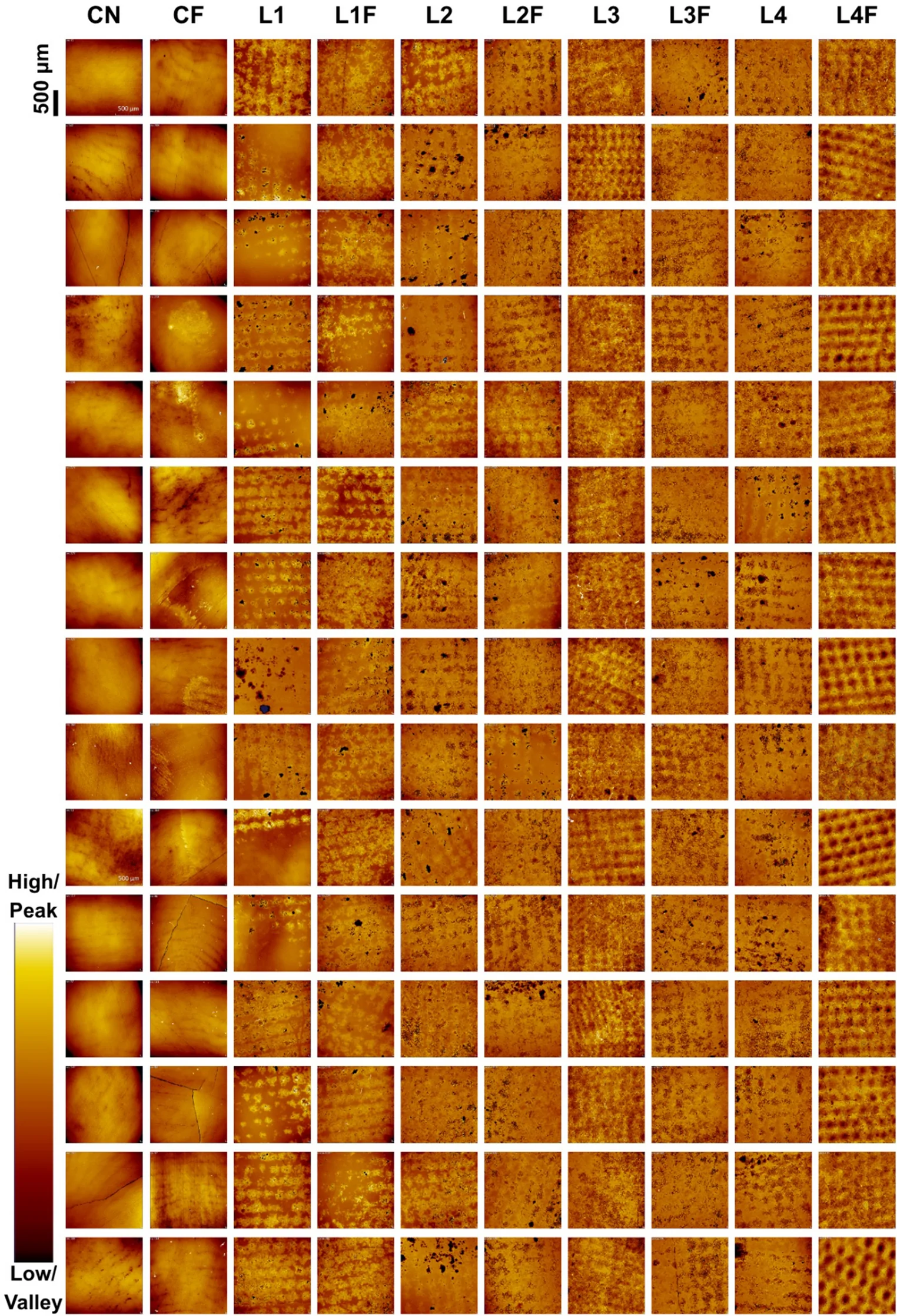
Profilometry 2D images (originals 1.5 × 1.5 mm). A representative relative lookup table is provided, where lighter colors (white/yellow) represent topographical peaks and darker colors (dark red/black) indicate valleys. Note that not all light areas correspond to higher surfaces, especially in the pulse regions, where the extreme irregularities of the melted enamel resulted in light scattering and optical artifacts (spikes)

The qualitative analysis of the 2D images (Fig. 1) and 3D profiles evidenced the progression of the surface alteration as more scans were performed. One or two laser scans (groups L1, L1F, L2, and L2F) did not ensure coverage of the entire area, leaving unaltered areas between pulses and between pulse rows. The increase to three and four irradiations expanded the extent of the modification, replacing the sound areas with a fused “base” layer over which the marks of the new pulses become evident.

**Fig. 2:**
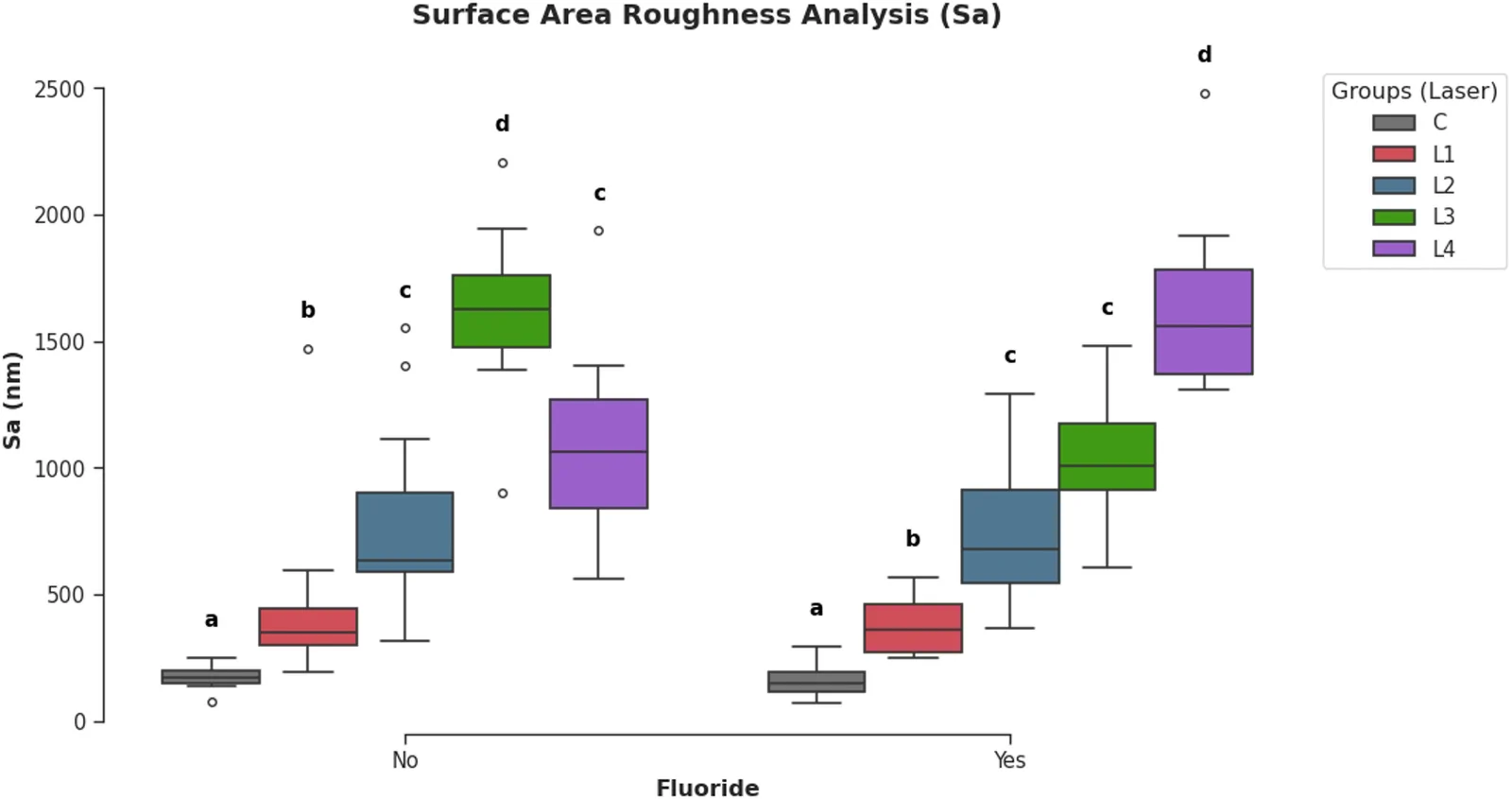
Analysis of "average roughness" or "deviation of all points from a plane fit to the test part surface" (Sa). Graph plotted using the original roughness values (nm). Different lowercase letters indicate statistically significant differences between groups (*p* < 0.05), while shared letters denote no significant difference, according to two-way ANOVA followed by Tukey’s post-hoc test

Quantitatively, a generalized increase in mean surface roughness (Sa) was observed, with a trend proportional to the number of irradiations (Fig. 2). The two-way ANOVA revealed a strong main effect for the laser scans (*p* < 0.001; η^2^_p_ = 0.846, indicating that the number of irradiations was the dominant factor, explaining approximately 85% of the variance in roughness) and a statistically significant interaction between irradiation and fluoride (*p* < 0.001; η^2^_p_ = 0.225). This interaction established the third irradiation as a threshold for surface alteration: it is from three irradiations onwards that a uniformly fused substrate is created over a significant area. Upon encountering this homogeneously modified surface, the application of fluoride (L3F) resulted in a homogenization of the topography, significantly reducing the roughness compared to L3 (*p* = 0.00032). In contrast, the fourth irradiation caused the re-melting of an already altered layer, creating an overlapping relief that, when interacting with fluoride (L4F), resulted in a significant increase in roughness compared to L4 (*p* = 0.00177).

### Scanning electron microscopy (melting)

**Fig. 3:**
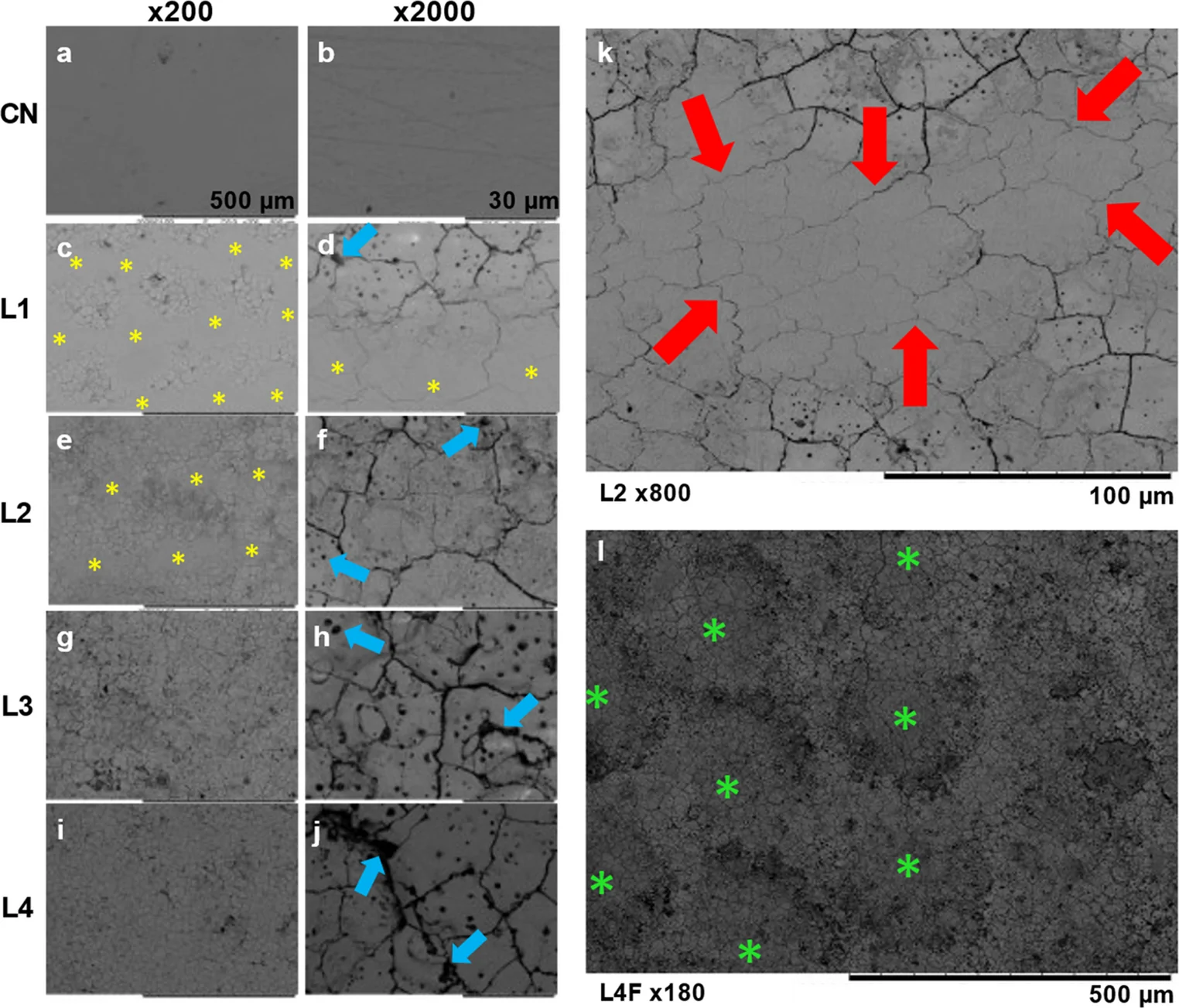
Representative Scanning Electron Microscopy images. The left panel standardizes the comparison across groups at x200 and x2,000 magnifications. Images (**c**) and (**e**) reveal the spatial distribution of individual pulses, with small yellow asterisks indicating unmelted enamel spaces between them. Groups L3 (**g**, **h**) and L4 (**i**, **j**) demonstrate comprehensive surface modification where the natural prismatic structure is replaced by amorphous, melted and resolidified layers (**h**, **j**). The right panel highlights specific phenomena: image (k, x800) details the thermal impact between pulses, with large red arrows pointing to microcracks in the interprismatic spaces; image (l, x180) exhibits pulses leaving marks (large green asterisks) over an already melted and resolidified surface, a pattern seen in the groups submitted to four scans. Small blue arrows are consistent with residual coal particles on the surface (d, f, h, j)

The microscopic images detailed the topographic findings and the magnitude of the thermal impact (Fig. 3), in addition to corroborating the patterns suggested by profilometry. At lower magnifications, the geometric individualization of the melting by the pulses was evident in groups L1, L1F, L2, and L2F, confirming the presence of unmelted spaces. At high magnification, it was possible to note that the enamel adjacent to the melting centers also suffered thermal modification, as microcracks propagating in the interprismatic spaces became clear. In groups L3, L3F, L4, and L4F, the surface appeared regularly modified, with a tendency toward the absence of unmelted spaces. The four-irradiation group exhibited an overlapping melting morphology, where the surface reached by the last sequence of pulses was already a previously fused and resolidified layer over its (apparent) entire extent. Additionally, blackened and irregular areas embedded in the modified surface indicated the retention of residual coal.

### ATR-FTIR (chemical/crystallographic modifications)

**Fig. 4:**
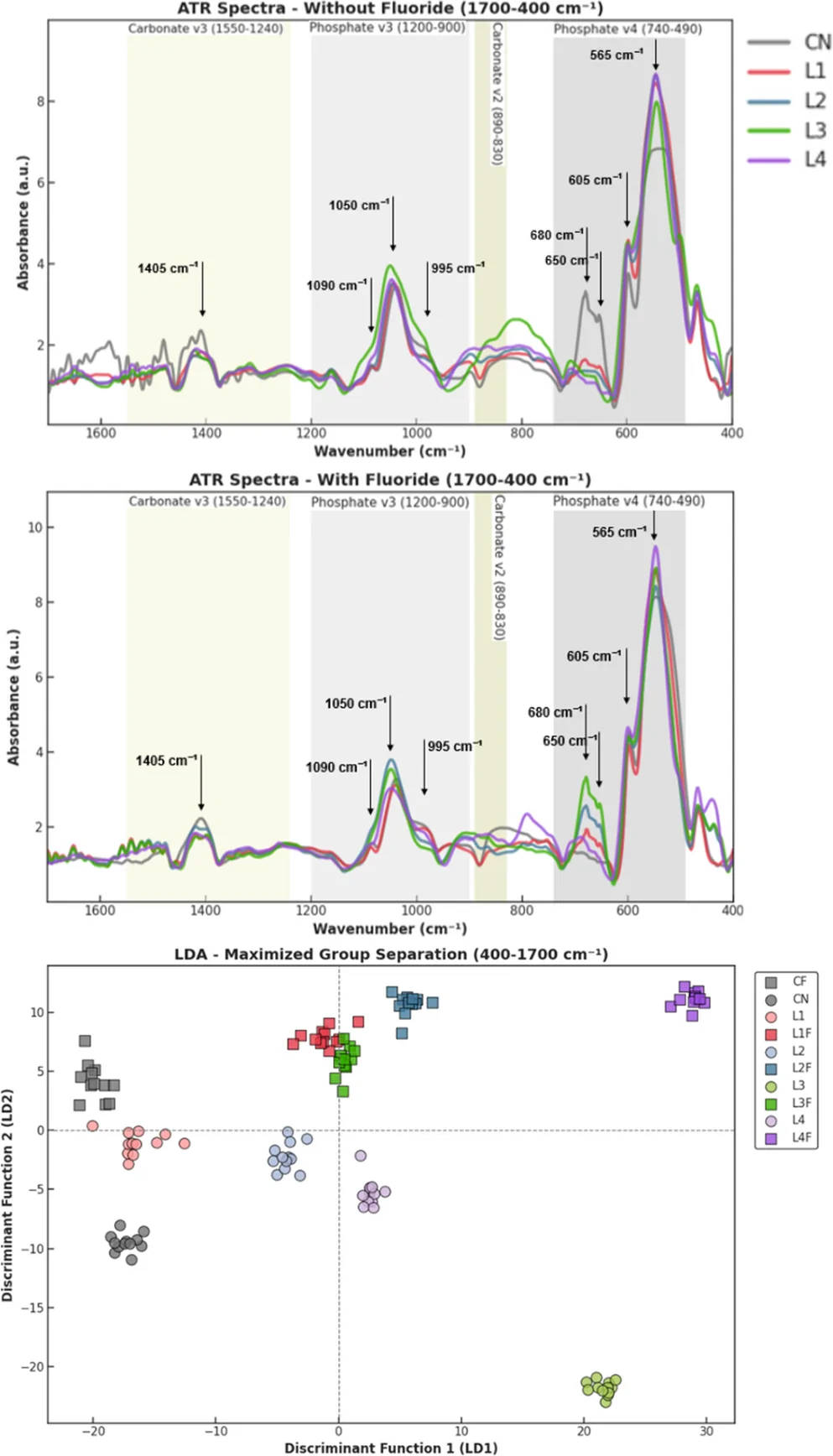
Composite image of FTIR spectra (top and middle) and multivariate analysis (bottom). Representative ATR-FTIR spectra of groups without and with fluoride, respectively, highlighting the primary carbonate (ν3, ν2) and phosphate (ν3, ν4) vibrational bands. Linear Discriminant Analysis (LDA) scatter plot derived from the mid-infrared spectral dataset (400–1700 cm⁻¹). The first linear discriminant (LD1) effectively separated the groups based on the number of irradiations (horizontal axis), while the second linear discriminant (LD2) effectively separated the groups treated with acidulated phosphate fluoride (above the horizontal dotted line) from those not treated (below the horizontal dotted line), demonstrating that both the laser thermal changes and the fluoride application yield distinct chemical signatures

To direct the investigation of the vibrational bands, the spectra from the selected region (400 to 1700 cm⁻¹) were initially subjected to Linear Discriminant Analysis (LDA) with leave-one-out cross-validation. The multivariate model segregated the treatments with an overall accuracy of 87.5% (Fig. 4). The analysis of the back-projection of the discriminant weights indicated that the separation of the groups was primarily guided by structural reorganizations in the phosphate (ν4) and carbonate (ν2 and ν3) bands, indicating that the spectral alterations reflect effective changes in the crystalline lattice and not merely morphological surface artifacts.

**Fig. 5:**
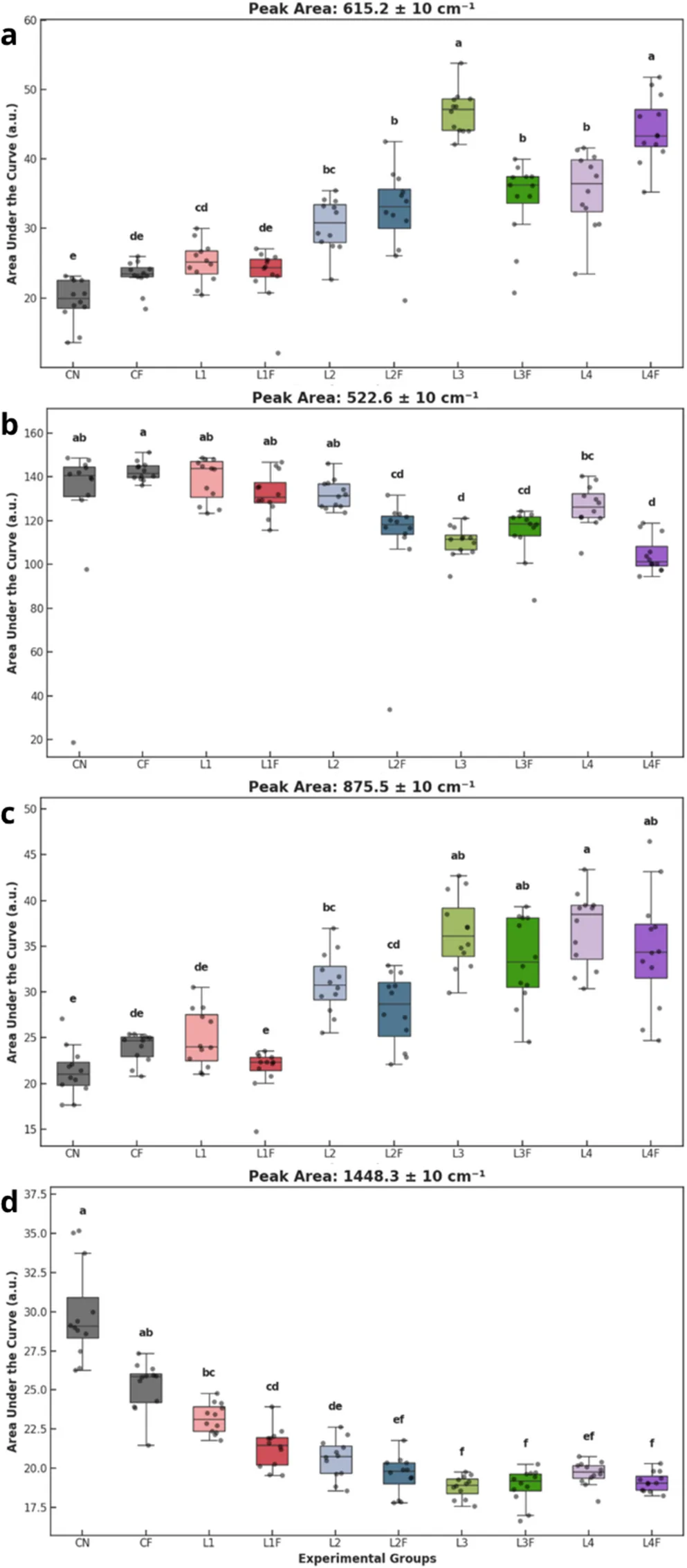
Area under the curve (ATR-FTIR) of four of the most influential bands in group separation according to multivariate analysis (LDA), associated to phosphate ν4 ~615.2 cm⁻¹ (**a**), phosphate ν4 ~522.6 cm⁻¹ (**b**), carbonate ν2 ~875.5 cm⁻¹ (**c**), and carbonate ν3 ~1448.3 cm⁻¹ (**d**). Different lowercase letters indicate statistically significant differences between groups (*p* < 0.05), while shared letters denote no significant difference, according to Tukey’s post-hoc test

For three of the four analyzed bands, the two-way ANOVA indicated a statistically significant interaction between laser irradiation and fluoride application, although the main effect of the number of irradiations was expressively greater than that of the APF application in all of them. In the band associated with the phosphate bending mode (ν4, ~615.2 cm⁻¹ - graph a, Fig. 5), a trend of increasing integrated area was observed as the number of irradiations increased. This region presented the strongest interaction of the study (*p* < 0.001; η^2^_p_ = 0.350) and a high influence of the isolated laser (η^2^_p_ = 0.761, indicating a large effect on the crystalline structural rearrangement). Groups L3 and L4F presented the highest medians, differing significantly from their respective non-laser controls (CN and CF) and their pairs with the opposite fluoride treatment (L3F and L4). Complementarily, the lower extremity of the main phosphate band (~522.6 cm⁻¹ - graph b, Fig. 5) exhibited an inverse behavior, with an area reduction in the irradiated groups. A significant interaction was also noted (*p* < 0.001; η^2^_p_ = 0.230), but with a greater effect from the irradiation (η^2^_p_ = 0.497), with groups L3 and L4F diverging the most from the controls.

To impartially quantify these alterations, the area under the curve (AUC) was calculated for the most relevant peaks highlighted by the model, using integration windows of ± 10 cm⁻¹ and a distance filter of 40 cm⁻¹ to avoid band redundancy. Four of these peaks, considered the most representative of the compositional changes in response to the applied treatments, were selected and are presented below (Fig. 5).

The impact of the treatment on carbonate, the soluble component of enamel, was quantified in its bending (ν2, ~875.5 cm⁻¹ - graph c, Fig. 5) and stretching (ν3, ~1448.3 cm⁻¹ - graph d, Fig. 5) modes. The peak area at 875.5 cm⁻¹ showed an increasing trend in the irradiated groups, reaching the highest statistical ranks in those subjected to 3 and 4 irradiations, presenting a smaller but significant interaction effect (*p* = 0.008; η^2^_p_ = 0.116) and a strong isolated effect of the laser (η^2^_p_ = 0.709). In contrast, the peak area at 1448.3 cm⁻¹ did not show a significant interaction between laser and APF (*p* > 0.05). The variance of this band was predominantly dictated by the strong isolated effect of the irradiation (η^2^_p_ = 0.788) and, to a lesser degree, of the fluoride (η^2^_p_ = 0.156), translating into a sharp decrease in all irradiated groups. The untreated control group (CN) exhibited the highest medians, confirming the thermal loss of the ν3 carbonate promoted by the laser irradiation.

## Discussion

The present study demonstrated that achieving a comprehensive modification of the enamel surface by the Nd:YAG laser depends on the number of successive irradiations (scans). By confirming that one or two scans leave unaltered spaces across the targeted area, the results emphasize the irradiation method as a critical variable. This physical evidence provides a plausible rationale that may help elucidate the divergences in efficacy mapped in the literature [8]. It suggests that the conflicting outcomes of protocols underestimating the irradiation technique – such as single scans or the omission of the photoabsorber [17–26] – could be related to an incomplete structural modification of the surface, in addition to the chosen laser parameters.

The topographic and melting pattern analysis (profilometry and SEM) of groups L1 and L2 revealed a heterogeneous surface, characterized by “islands” of unaltered enamel interspersed with fused regions. This can be explained, besides the Gaussian distribution of the beam, by an interaction with the photoabsorber. Since the absorption of Nd:YAG laser radiation by sound enamel is minimal, the thermal interaction depends crucially on the photoabsorbing substance [10, 13, 14]. The pattern of spaces between pulses suggests that the coal suspension undergoes vaporization exactly where it receives the pulse impact. Consequently, when the collimated beam advances and the subsequent pulse strikes a part where the photoabsorber has already been vaporized, the temperature increase in this small area is no longer the same as it would be over an intact photoabsorber. From this physical perspective, protocols described in the literature [28, 29] that employ consecutive manual irradiations, but without reapplying the photoabsorber, lose the foundation of their mechanism of action.

Considering that, even when using a motorized stage strictly parameterized to ensure pulse overlapping, unaltered areas were observed in the groups with fewer scans, manual clinical application will certainly result in an even more flawed modification, as reported [16]. Considering that the preventive effect of the laser depends on the physical-chemical and structural transformation of the entire targeted area, manual clinical application tends to be even more irregular and inefficient. In this context, reporting manual irradiation by time is inaccurate because it depends on the target area and the personal application speed. Considering the physical interaction and motor limitations, it would be more beneficial to recommend for all cases: irradiating at the lowest possible speed over the entire surface of interest to completely or largely consume the photoabsorber; and repeating this process three times (including the photoabsorbing substance before each one).

Overcoming this limitation in this in vitro model occurred by repeating the scans with the reapplication of the coal suspension. This protocol established a clear threshold for surface alteration (L3) (Figs. 1 and 3g, h), in which a uniformly fused surface was observed. It is worth noting that the uniformity cited here refers to the fused area and not to a surface with low roughness or equally melted. The clinical importance of reaching this threshold was evidenced in the synergistic interaction with topical fluoride suggested by the roughness (Sa) data. While the isolated irradiation (L3) generated a rough surface, the subsequent application of APF (L3F) on this substrate promoted a reorganization that smoothed the topography, significantly reducing the mean surface roughness. In contrast, the fourth irradiation (L4) caused the re-melting of an already structurally altered tissue, creating an overlapping relief (Fig. 3l) that, upon receiving fluoride (L4F), elevated the Sa values.

The increase in roughness in all irradiated groups indicates that the adoption of multiple scans imposes a strict balance on dental practice: even though repetition is mandatory to ensure structural uniform changes and thus acid resistance, the process results in an inevitable increase in surface roughness compared to polished enamel. Although rough surfaces are favorable niches for the adhesion and retention of cariogenic biofilm, and it may seem that the increase in roughness will result in an increased caries index, previous studies indicate that this does not happen because the physically and chemically altered substrate is associated with higher resistance to acid dissolution [7, 30].

Beyond the clear thermal alteration of melting, the high magnification in SEM drew attention to the adjacent thermal impact. The presence of microcracks propagating in the interprismatic spaces in the peripheral regions to the melting centers (Fig. 3k) suggests that the laterally transmitted heat denatures the organic matrix and vaporizes structural water even in zones that did not reach the melting temperature. This finding makes it explicit that unmelted areas are not necessarily unaltered by irradiation, but result in a heterogeneous coverage that impacts fundamental properties of the enamel: the loss of structural water between 200 °C and 400 °C can lead to ionic substitutions that result in a more stable structure [31]; the decomposition of proteins between 350 °C and 400 °C increases enamel permeability [8] and affects the enamel’s ability to dissipate stresses [32], but decreases the soluble part of carbonate at 100 °C to 800 °C [33].

Chemically, the high temperatures induced by the multiple scans promoted a reorganization of the crystalline lattice, confirmed by the quantitative analysis of the ATR-FTIR spectra. The increase in the integrated area of the phosphate ν4 band (~615.2 cm⁻¹) in the irradiated groups (especially L3 and L4F) may indicate a higher phosphate content in the crystals. This change in the hydroxyapatite lattice is spectroscopically corroborated by the reduction in the area at the base of the phosphate band (~522.6 cm⁻¹). As established in the literature [34, 35], the vibrational spectroscopy of apatitic mineral phases establishes that crystallization and the increase in crystal size result in narrower absorption bands (smaller full width at half maximum - FWHM), with a consequent loss of area at the extremities of the band. The significant interaction effect (Laser x Fluoride) in these peaks suggests that the irradiation creates a thermally modified surface that acts favorably towards ionic rearrangement and crystalline order upon the subsequent application of fluoride.

The quantitative analysis of the integrated areas (Fig. 5) statistically corroborates these structural changes. Specifically, the drop in the carbonate ν3 band area (1448.3 cm⁻¹, Fig. 5d) across irradiated groups indicates thermal decarbonation. Considering the localized nature of the laser pulse, it is physically logical that this decarbonation occurs from the first scan. The downward trend in Fig. 5d strictly reflects the physical expansion of the altered area rather than an intensified per-pulse chemical alteration. Because the ATR crystal acquires data from random microscopic footprints, samples with one or two scans retain areas not fully altered between pulses. Consequently, the crystal frequently reads high-carbonate unfused enamel, driving the average area detection upward for the L1 and L2 groups. In contrast, protocols with three or four irradiations ensure comprehensive surface transformation, explaining the stabilization of the AUC readings in the L3 and L4 groups and demonstrating that uniform chemical alteration depends on the scanning technique.

Regarding the components most soluble to acid demineralization, the spectroscopic evaluation indicated the occurrence of thermal decarbonation. The expressive drop of the band at ~1448 cm⁻¹ (ν3) reflects a decrease in the overall amount of carbonate in the surface inorganic matrix [36]. The localized increase detected at the peak of ~875.5 cm⁻¹ (ν2) suggests a reorganization of the mineral, with an increase or repositioning of the carbonate in the crystalline lattice [37]. The elimination of carbonate associated with the increased organization of phosphates is aligned, from a crystallographic/chemical point of view, with the preventive potential of the therapy with multiple Nd:YAG scans reported in the clinical literature.

After the 4-minute APF application and removal of excess gel, the specimens were left undisturbed for 30 minutes before rinsing, and this was designed to maximize its acidic etching effect, facilitating the observation of surface roughness changes. Clinically, however, the gel is subjected to dynamic salivary flow, which would likely reduce the magnitude of the roughness alterations observed in this study. 

Finally, the limitations of this in vitro model must be acknowledged. Sample preparation required flattening and polishing, exposing a subsurface enamel that differs compositionally from the natural intact outer surface. Furthermore, the absence of intraoral dynamics (salivary flow and mechanical wear) and a direct cariogenic challenge (such as pH cycling) limits direct inferences regarding caries resistance. Nevertheless, the comprehensive morphological and chemical modification across the entire area, achieved exclusively through multiple scans, mechanistically supports the preventive clinical success previously reported for this specific protocol [7], elucidating why simplified single-scan irradiations may fail to yield protective effects.

## Conclusion

A single irradiation with the Nd:YAG laser is incapable of uniformly modifying the enamel surface, resulting in localized and spaced alterations, even under automated irradiation. Our results demonstrate that the use of multiple successive scans (a minimum of three), with the mandatory reapplication of the photoabsorber, is essential to ensure that the entire treated area is modified. In conclusion, future in vitro and clinical studies must prioritize the irradiation technique (specifically multiple scans with photoabsorber reapplication) as a primary variable for achieving effective and reproducible enamel modification, just as strictly as standardized laser parameters.

## Data Availability

The datasets generated during and/or analysed during the current study are available from the corresponding author on reasonable request.
